# A Review of Inflammatory Bowel Disease: A Model of Microbial, Immune and Neuropsychological Integration

**DOI:** 10.3389/phrs.2021.1603990

**Published:** 2021-05-05

**Authors:** P. Tavakoli, U. Vollmer-Conna, D. Hadzi-Pavlovic, M. C. Grimm

**Affiliations:** ^1^ St George and Sutherland Clinical School, Sydney, NSW, Australia; ^2^ School of Psychiatry, University of New South Wales, Sydney, Australia

**Keywords:** IBD, neuropsychology, immunology, microbiome, physiology, autonomic nervous system, IBD therapeutic

## Abstract

**Objective:** Inflammatory bowel diseases (IBDs) are complex chronic inflammatory disorders of the gastro-intestinal (GI) tract with uncertain etiology. IBDs comprise two idiopathic disorders: Crohn’s disease (CD) and ulcerative colitis (UC). The aetiology, severity and progression of such disorders are still poorly understood but thought to be influenced by multiple factors (including genetic, environmental, immunological, physiological, psychological factors and gut microbiome) and their interactions. The overarching aim of this review is to evaluate the extent and nature of the interrelationship between these factors with the disease course. A broader conceptual and longitudinal framework of possible neuro-visceral integration, core microbiome analysis and immune modulation assessment may be useful in accurately documenting and characterizing the nature and temporal continuity of crosstalk between these factors and the role of their interaction (s) in IBD disease activity. Characterization of these interactions holds the promise of identifying novel diagnostic, interventions, and therapeutic strategies.

**Material and Methods:** A search of published literature was conducted by exploring PubMed, EMBASE, MEDLINE, Medline Plus, CDSR library databases. Following search terms relating to key question were set for the search included: “Inflammatory bowel diseases,” “gut microbiota,” “psychological distress and IBD,” “autonomic reactivity and IBD,” “immune modulation,” “chronic inflammation,” “gut inflammation,” “enteric nervous system,” “gut nervous system,” “Crohn’s disease,” “Ulcerative colitis”, “depression and IBD”, “anxiety and IBD”, “quality of life in IBD patients,” “relapse in IBDs,” “remission in IBDs,” “IBD disease activity,” “brain-gut-axis,” “microbial signature in IBD,” “validated questionnaires in IBD,” “IBD activity indices,” “IBD aetiology,” “IBDs and stress,” “epidemiology of IBDs”, “autonomic nervous system and gut inflammation”, “IBD and environment,” “genetics of IBDs,” “pathways of immune response in IBDs,” “sleep disturbances in IBD,” “hypothalamic-pituitary-adrenal axis (HPA),” “sympatho-adrenal axis,” “CNS and its control of gut function” “mucosal immune response,” “commensal and pathogenic bacteria in the gut,” “innate and adaptive immunity.” Studies evaluating any possible associations between gut microbiome, psychological state, immune modulation, and autonomic function with IBDs were identified. Commonly cited published literatures with high quality research methodology/results and additional articles from bibliographies of recovered papers were examined and included where relevant.

**Results:** Although there is a substantial literature identifying major contributing factors with IBD, there has been little attempt to integrate some factors over time and assess their interplay and relationship with IBD disease activity. Such contributing factors include genetic and environmental factors, gut microbiota composition and function, physiological factors, psychological state and gut immune response. Interdependences are evident across psychological and biological factors and IBD disease activity. Although from the available evidence, it is implausible that a single explanatory model could elucidate the interplay between such factors and the disease course as well as the sequence of the effect during the pathophysiology of IBD.

**Conclusion:** Longitudinal monitoring of IBD patients and integrating data related to the contributing/risk factors including psychological state, physiological conditions, inflammatory/immune modulations, and microbiome composition/function, could help to explain how major factors associate and interrelate leading to exacerbation of symptoms and disease activity. Identifying the temporal trajectory of biological and psychosocial disturbances may also help to assess their effects and interdependence on individuals’ disease status. Moreover, this allows greater insight into understanding the temporal progressions of subclinical events as potential ground for disease severity in IBD. Furthermore, understanding the interaction between these risk factors may help better interventions in controlling the disease, reducing the costs related to disease management, further implications for clinical practice and research approaches in addition to improving patients’ mental health and quality of life.

## Inflammatory Bowel Diseases

### Introduction

Inflammatory Bowel Diseases (IBDs) are debilitating chronic inflammatory disorders of the gastrointestinal tract with the peak age of onset in adolescence and young adulthood [1]. IBDs are complex chronic inflammatory disorders with multiple factors such as psychological distress, autonomic dysfunction, gut microbiome dysbiosis and immune modulations associated with disease activity. The incidence of IBDs has shown an increasing trend over the last few decades. This was initially noted in Western countries and is now being increasingly observed in countries such as Japan, China and India [2]. IBDs are recognized as a serious, worldwide public health issue with an increasing economic and financial burden. This review has several key aims including 1) to present an overview on IBDs, the disease course and immune modulations in IBD, 2) to outline the importance of the autonomic nervous system and its possible association with chronic inflammatory conditions such as IBDs and their symptomatology, 3) to examine the impact of psychological factors and IBD, and 4) to address the role of gut microbiota and its interplay with disease activity.

Inflammatory bowel diseases: IBDs are characterised by chronic inflammation of the GI tract and comprise two idiopathic gastrointestinal disorders known as ulcerative colitis (UC) and Crohn’s disease (CD). Despite intense research efforts, the disease aetiology (ies) is (are) not fully understood. However, it appears that both genetic and environmental factors are involved in IBD causation, affecting the interaction between the intestinal mucosa and luminal bacteria, with a breakdown in the regulatory constraints of mucosal immune responses to enteric bacteria. In other words, an immune (inflammatory) response that is too easily triggered and/or needlessly prolonged. Both UC and CD are chronic disorders of a remitting and relapsing kind. Just as the cause of the initial onset of IBD is unknown, what leads to remission and relapse is also uncertain [3].

The incidence of inflammatory bowel diseases is increasing globally and in 2012, the highest reported prevalence values for IBD were in Europe (UC, 505 per 100,000 persons; CD, 322 per 100,000 persons) and North America (UC, 249 per 100,000 persons; CD, 319 per 100,000 persons) [4]. It has been estimated that in developed countries up to 360 in every 100,000 individuals have IBD [5]. The incidence of CD has increased by 70% and incidence of UC has increased by 60% [4] with an increasing trend in Asia since 1988 [6]. There is a high incidence of inflammatory bowel diseases in Australia [7–10]. According to an estimate by Gastroenterological Society of Australia in 2018, Australia has one of the highest prevalence rates of IBD worldwide with approximately 75,000 Australians diagnosed with IBD and over 1,622 new cases diagnosed every year, 776 with CD and 846 with UC (https://www.gesa.org.au/public/13/files/Clinical%20Updates%20and%20Guidelines/2018_IBD_Clinical_Update_May_update.pdf).

UC is characterised by chronic inflammation of the large intestine with abnormal activation of the immune system. It affects the inner-most layer of the colon and rectum [11]. CD can affect any level of the intestinal tract from the mouth to the anus and across all layers of the bowel wall, but mostly affects lower small intestine (ileum) and colon. The most common symptoms of IBD include diarrhea, rectal bleeding, intermittent nausea and vomiting, and abdominal pain or tenderness [12–14]. The symptoms are due to intestinal damage resulting from the exaggerated inflammatory response. Complications from these immune-mediated diseases include anemia, malnutrition, bowel obstruction, fistula, infection, and an increased risk of colon cancer. Extra-intestinal manifestations may also develop, such as joint problems (arthralgia, arthritis, and ankylosing spondylitis), rashes and skin conditions (erythema nodosum, psoriasis), chronic liver disease (primary sclerosing cholangitis) and eye conditions (such as uveitis). There is no cure for IBD. Clinical management focusses on keeping patients in remission and asymptomatic with a primary aim of reducing inflammation during relapse and secondary aims of prolonging the time spent in remission and mucosal healing.

#### Contributing Factors in IBDs

IBDs are complex diseases with multiple factors likely to contribute to the aetiology and pathogenesis of the diseases. There is an accumulating body of research exploring potential factors thought to contribute to the etiology of IBDs. Over the last 50 years, but mainly in the last two decades, various advances have brought attention to the mechanisms, and interplay of the underlying factors in IBD disease activities and its progression. Included in these are increased knowledge of cell and molecular biology, deeper insight into the immune response and potential immune cascades in chronic inflammatory disorders as well as breakthrough technologies in genetic analysis of diseases. It is worth highlighting the innovation of genome wide association studies and subsequent omics analyses, together with understanding of the function of gut microbiota in health and disease, as significant advances improving our understanding of disease development. It seems that there is a need to apply a combined approach in studying IBDs to have better understanding of the whole orchestra of complex contributors to the disease, including genetics, as well as microbiome-related, dietary-related, autonomic function, environmental and psychosocial factors, and their longitudinal interplay.

##### The Role of Genetics in IBDs

What we know about genetic predisposition in IBDs is largely based upon comparative studies that investigated the prevalence of such diseases in first degree family members, relatives of the sufferers as well as on twin studies. These genetic studies have greatly increased our understanding of the pathogenesis of IBDs. The prevalence of IBDs is higher in the relatives of IBD patients [15]. In 1980, Mayberry looked in to the prevalence of Crohn’s disease in families of IBD patients and estimated a 30 fold higher risk of developing IBD in a sibling compared with the normal population risk [16]. Monsen also identified a higher prevalence of UC in first degree family members compared to the general population [17]. Similarly, in twin studies of CD and UC, a strong familial aggregation has been observed in monozygotic twins [15, 18]. Genome-wide association studies (GWAS) have been helpful in identifying hundreds of genetic variants in Crohn’s disease and ulcerative colitis [19, 20].

Genotyping studies of IBDs highlighted considerable heterogeneity between, and within, UC and CD, with some genes common to both and some separate. So far more than 200 gene polymorphisms and genetic variants have been identified that are associated with IBDs [21, 22]; many of these genes increase the risk of the development of disease by only a very small amount. Furthermore, approximately 70% of IBD risk loci are shared with other immune-mediated disorders including type 1 diabetes mellitus, celiac disease, rheumatoid arthritis, ankylosing spondylitis, and psoriasis [23]. One-third of loci described confer susceptibility to both CD and UC [24].

The first gene identified to be associated with Crohn’s disease was Nucleotide-binding oligomerization domain-containing protein 2 (NOD2). This gene is important in immune stimulation against bacterial invasion [25]. Many other IBD risk alleles are associated with either host response to bacterial invasion, development of adaptive or innate immunity, autophagy pathways, phagocytosis, or mucosal barrier function [3]. Disruption in the function of many of these genes increases the risk of development of inflammatory disorders in addition to IBD. Despite the importance of genetic predisposition, there is no single genetic variant that can explain the fast trend development of IBDs. It is also not clear why most individuals, who carry IBD-associated risk variants, remain healthy while others develop IBD or even develop more than one immune-mediated disease. Clearly, genetics alone cannot explain the susceptibility and progression of IBDs. Therefore, the study of the characteristics and roles of other factors that may interact with genetic vulnerabilities in patients with IBDs is critical.

##### IBDs and Environmental Factors

There is a wide spectrum of different environmental factors proposed to influence the etiology and disease activities of chronic inflammatory disorders such as IBDs. Population based studies have proposed higher incident of IBDs in urban than in rural populations [26, 27]. Evidence supports the inverse association between risk of IBDs with early life exposure to farm animals, pets, larger family size, greater number of siblings [28, 29], mode of childbirth [30] and breast feeding [31]. All such early exposures are known as important determinants for more diverse gut microbiome in early life as an internal environmental factor [29, 32, 33]. The beneficial interaction between gut microbiome with host genetics and immune system is an important internal environmental factor in disease pathogenesis although, external environmental factors are also strong determinants of health and disease. Diet is a major contributor and has both short-term and long-term influence on intestinal microbiome which can have influence on risk of incidence of the diseases [34, 35]. Many studies that examined the influence of diet on the course of the disease suggested the strong association between the two [36–39].

While studies have shown the increase in risk associated with smoking in CD [40], current smokers have shown strong inverse risk association between smoking and UC [41–43]. Smoking is associated with more severe disease course, greater risk of immunosuppression use or surgery and more risk of recurrence after surgery in CD patients whereas in UC, smoking cessation is commonly a precipitant for relapse within a year of cessation, current smokers have fewer surgeries, milder disease course and less need for immunosuppressive drugs [44–46]. Studies proposed the divergent effect by appendicectomy on UC suggesting inflammation of appendix might have protective interplay with the disease [47, 48]. Early childhood perturbation of gut microbiota through antibiotic therapy has been proposed to alter the gut immune response and influence the susceptibility to IBDs [49, 50].

One of the most significant current discussions in studying complex diseases is the concept of modernised or westernised lifestyle which associates chronic and complex disorders such as IBDs with rapid industrial changes and their effect on the environment and social behaviour [51]. Decline in the incidence of infectious diseases in developed countries due to improved hygiene standards and infection prevention methods (hygiene hypothesis) [52, 53], has resulted in loss of initial symbiotic relationship between the human host and microorganisms [54]; that mutualism is considered essential in development of human immune responses. High levels of stress as a common constituent of industrialisation and its effect on the immune response, neuroendocrine disturbances and psychological adaptation are of major concern in pathophysiology of chronic and complex inflammatory disorders.

##### Why Consideration of Psychological Variables in IBD is Important

It is well documented that any chronic disease is associated with a greater burden of psychological stress, depression, anxiety and altered quality of life [5, 55–59]. IBD follows the same model of neuropsychiatric co-morbidities, which are more prevalent during an active disease when the disease is difficult to control [60]. Although it could be projected that illness leads to psychological problems through a uni-directional effect on patients’ wellbeing and quality of life, bidirectional interplay between disease factors including systemic and local inflammatory activities and the brain is now a generally accepted hypothesis. The study published in 2018 similarly supported this idea when they observed high anxiety scores in patients with clinically active IBD compared to the time these patients were in clinical remission. Same study suggested patients with quiescent disease activity at baseline but abnormal anxiety score had higher rate of relapse when followed up or needed escalation of their therapy [61]. Psychological states can influence patients’ behaviour and their perception of the disease through different neuro-endocrine pathways [62, 63]. The role of stress - conceptualised both as an environmental/psychosocial challenge, as well as an internal stressor such as an evolving illness—has been investigated in the course of IBD, and it has been shown that stress can aggravate physiological, psychological and environmental vulnerabilities leading to emotional distress and potentially onset of mental and physical disorders [5]. It has been proposed that depression and anxiety are more prevalent in IBD patients with greater associations with IBD disease activity [64, 65]. Depression as a neurobehavioral factor can influence the risk of IBD as a pre-existing contributor, it also can be simply a product of the IBD as debilitating disease [66]. Depression can potentially influence the course of the disease by mediating through autonomic nervous system, predominantly through sympathetic nervous system [67, 68].

Nevertheless, studies on causality of psychological states and IBD symptoms have led to contradictory conclusions [64, 69]. Research has identified some aspects of psychological distress commonly manifested in IBD patients including depression and anxiety [70–73] other investigators did not find any evidence of association between psychiatric disorders and UC/CD disease activity [74, 75]. Some data suggested that both depression and anxiety preceded UC (and not CD) [76], while other literature indicated IBD was not a product of psychological distress [77, 78].

##### Physiological Factors and Their Role in IBDs

Digestive system functions and homeostasis are modulated through both central (CNS) and enteric nervous system (ENS) influences. Bayliss in 1899 proposed innervations in small intestine and their ability to generate downward movements including swaying motion (known as pendulum movement) accompanied by waves of longitudinal constriction, [79]; the exploration of the remarkable functions of an ENS developed further through the mid-20th century [80], described how new technologies and their applications facilitated our understanding of ENS’s complexity and neural connectivity in gut [80]. The bidirectional communication of brain and gut is facilitated by means of various pathways. Brain to gut signaling is through hypothalamic-pituitary-adrenal axis (HPA), and two branches of autonomic nervous system (ANS), sympatho-adrenal axis and descending monoaminergic pathways (spinal reflexes) [81]. Gut to brain signaling (bottom-up signalling) is via primary visceral afferent neurons (extrinsic including vagal and spinal afferent as well as intrinsic afferent neurons), enteroendocrine signaling and immune related signaling to brain [81].

The sympathetic nervous system (SNS) modulates GI function and immune regulation by the close proximity of nerve endings to immune cells such as dendritic cells, B lymphocytes and mast cells [82]. The parasympathetic innervations of GI tract (vagal and sacral parasympathetic divisions) are thought to have an anti-inflammatory, immune modulatory role. An example of such activities is the modulation of macrophage activation through nicotinic acetylcholine receptors [83]. Alterations or dysfunction in the activities of the autonomic nervous system (perhaps related to stress, anxiety and depression) alters autonomic output to the gut and is likely to affect brain-gut signaling, gut function and its immune regulation [5].

##### Gut Microbiota and IBDs

Study of the gut microbiota composition and its function has become a major area of interest within the field of gastrointestinal disorders including inflammatory bowel diseases [84–86]. Many studies have addressed the possible link between gut microbiota and a maladaptive immune response in genetically susceptible and environmentally at-risk individuals [87–89]. The inappropriate immune response could be either translated to failure in tolerating the commensal bacteria or immune activation toward altered microbial populations (so-called dysbiosis) or function. Although a considerable literature has grown up around the theme of microbiota and inflammatory bowel diseases, there is inadequate knowledge about whether alterations in gut microbiota play a primary role in development of IBDs or are simply a consequence of chronic inflammation [85, 90–92].

### Immune Modulation and Inflammatory Bowel Disease

#### Introduction

The human immune system plays a critical role in recognition, response and adaptation to countless self and foreign molecules, so its integrity is very important in maintaining and recovering health. There are complex innate and adaptive mucosal immune mechanisms in the gastrointestinal tract that regularly examine the luminal contents and can detect microbial or food antigens and can activate immune pathways. Both innate and adaptive immune mechanisms are integrated with different mediators and immune cells to maintain tolerance, manage low grade inflammation and upregulate during the active phase of gut pathologies, including IBDs [93].

Cells of the immune system including dendritic cells, B cells and macrophages are considered as antigen presenting cells (APCs), which are important in both innate and adaptive immunity and immune homeostasis and are able to secret cytokines and activate innate immunity as well as presenting antigens to cells of the adaptive immune system, therefore relating adaptive and innate immunity pathways [94, 95].

Innate immunity comprises programmed and automatic defence-related elements such as the mucosal barrier, tight junctions of the epithelial cells and control of gut permeability, and secretion of antimicrobial enzymes such as defensins and lysozyme to protect the lamina propria from microbial raids [96]. Cells of the innate immune system form one of the nonspecific defense mechanisms against any invasions and include: macrophages, monocytes, neutrophils and other granulocytes, natural killers (NKs), dendritic cells, mast cells, and innate lymphoid cells (ILCs). Non-immune cells also play important roles: intestinal epithelial cells (IECs), endothelial cells, transforming growth factor-β (TGF-β) releasing stromal cells and mesenchymal cells. Epithelial cells contain and are coated with pattern recognition receptors (PRRs: Toll like receptors–TLRs–and Nucleotide-binding Oligomerization Domain-like receptors–NOD like receptors), which are a key component of the innate immune system and can recognise common repetitive patterns present on Gram-positive and Gram-negative bacteria, viruses, parasites, and fungi [97].

These receptors are sensitive to pathogen associated-molecular patterns (PAMPs) such as bacterial lipopolysaccharides (LPS) [98], Gram-positive and mycobacterial lipopeptide [99], lipoteichoic acid (LTA) and peptidoglycan (PGN) [100], double stranded viral RNA and bacterial DNA [101], and reactive oxygen species (ROS) induced by commensals [102]. Toll like receptors (TLR) are expressed by IECs in small intestine and colon [103, 104]. Signalling from TLRs leads to epithelial cell proliferation, safeguarding of IEC tight junctions, release of IgA and expression of antimicrobial peptides, regulation of proinflammatory cascades through signaling lamina propria immune cells [105, 106], and production of inflammatory cytokines, in case any products of the bacteria permeate the epithelial layer and are sensed by these receptors [107]. Nucleotide oligomerization domain (NOD1 and NOD2) are additional pattern recognition receptors that are intracellular and are required for defence against invasive enteric pathogens [108]. NOD2 is a primary receptor for muramyl dipeptide (MDP)-an immunoreactive peptide constituent of the cell wall of all gram positive and gram-negative bacteria. This peptide plays an important role in bacterial cell integrity and delineate the bacterial cell shape. Sensing of this peptide by the NOD2 receptor results in secretion of antibacterial peptides like α-defensins from the epithelial cell layer [109].

Activation of PRRs in columnar epithelial cells commonly leads to stimulation of transcription factor NF*k*B, a protein in cytoplasm of epithelial cells and nearly all cell types [110] that controls transcription of DNA in the nucleus of the cells, and cytokine production as part of its role in innate immunity as well as many other conserved cellular functions related to cell survival, cell growth and development, and apoptosis. Activation of NF*k*B may occur through various activation mechanisms [111] and eventually initiates a prototypical proinflammatory signaling in intestinal inflammation [112]. Activation of NF*k*B results in upregulation of proinflammatory factors including adhesion molecules, TNF-α, IL-1, IL-6 and IL-8. These molecules control and regulate immune responses and are important in recruiting leukocytes to the inflamed area [111].

Adaptive immunity presents specific immune responses either through B cell or T cell antigen-specific activation. This immune system covers antibody-mediated immune response (adaptive humoral immunity) and adaptive cell mediated immunity; critical contributors include cytotoxic T cells, effector T cells, regulatory T cells (Tregs), T helper lymphocytes and antibody-secreting B cells. Immune cells of the adaptive immune system are differentiated in Peyers’ patches of the small intestine, lymphoid follicles of the colon, or mesenteric lymph nodes [113].

The basic development of the function of the human immune system depends on its interaction with the human microbiome [114]. In all mammals the intestinal lumen is colonised by communities of bacteria that result in biofilm production-the proteolytic cleavage of the outer coating of mucus, which creates a barrier to infection. Any challenge that alters the microbiota composition and suppresses their regrowth can disturb the barrier to allow infection and disease [115]. The thickness of this mucus layer of the epithelium is correlated to bacterial content of the intestine; it is thinner in the proximal small intestine (containing 10^3^–10^5^ organisms per gram) and is thicker in the distal small and large intestine (containing 10^10^–10^12^ organisms per gram). Beneath and between the IECs, the lamina propria is the home of stromal cells, T cells, dendritic cells, macrophages, B cells (IgA producing plasma cells), and intraepithelial lymphocytes. Dendritic cells of lamina propria establish tight junction like structures with epithelial cells to have direct bacterial uptake from the intestinal lumen [116]. They are positioned to reach into the lumen and sample the luminal contents as an important surveillance strategy [117]. Activated dendritic cells then migrate to lymph nodes where they can activate T cells.

#### Immune Modulation and IBDs

Studies on inflammatory bowel diseases have revealed evidence of breakdown in immune regulatory constraint [116] and immune modulation in active phases of the diseases [118, 119]. IBD is generally believed to be driven by an increased population of effector T cells and increased production of proinflammatory cytokines (such as TNF-α, IL-6, IFN Ɣ). The balance between proinflammatory and immunosuppressive forces can determine the progression of inflammation [120, 121]. It has been suggested that epithelial barrier damage could lead to increased intestinal permeability and the influx of intestinal lumen microbes and antigens into the underlying layer including lamina propria, which is the home for immune cells and mediators [96]. Hence, it seems that immune upregulation in IBDs is an outcome of barrier damage and dysregulated cellular responses, with consequent instigation and progression of inflammation in the lamina propria.

The complex orchestrated cross talk between the immune activation and cytokine secretion, stimulates naive T cells (homing in peripheral lymphoid tissues and lymph nodes) leading to proliferation and activation of effector T cells and memory T cells. Stimulated effector T cells migrate to intestinal lamina propria and nearby blood and lymphatic vessels. Here, lymphocyte cell adhesion molecules (selectins and integrins), and their ligands located on endothelial cells, mediate homing of effector cells. Activated macrophages stimulate adaptive immunity locally and activated dendritic cells migrate to nearby lymphoid tissue where they arouse a range of T helper cells (CD4^+^) and cytotoxic T cells (CD8^+^) as well as assisting the maturation of regulatory T cells. Th17 cells are important in defense against mucosal microbial and fungal infections. The key inducing cytokines for activation of Th17 pathways are IL-1, IL-6, IL-23 and transforming growth factor β (TGF-β). Activated Th17 cells release the proinflammatory IL-17 family of cytokines, which are important in the pathogenesis of human colitis [122, 123]. IL-17 is an important cytokine for recruitment of Treg cells and neutrophils [124]. IL-23 is the principal cytokine that regulates the maintenance and function of Th17 cells. In murine studies, it has been shown that IL-6, TGF-β, IL-1β, IL-23 and the ATP that derives from commensal bacteria (such as segmented filamentous bacteria) are required for Th17 cell differentiation. Recent studies have implicated immune modulation by the microbiota as it has been shown that germ free animals have impairment of Th17 cell development and reduced levels of IL-17 production in the colon [125]. Also, Treg cells in these animals are not as effective as in conventionally colonised animals [126]. An interesting and repeated finding is that in spite of high concentration of anti-inflammatory cytokines such as TGF-β and IL-10 during the active phase of IBDs, mucosal inflammation persists, which underlines deficient immune regulation and complexity of the immune pathways involved in IBDs [127, 128]. Treg cells regulate the level and function of the proinflammatory cytokines derived from effector T cells, and can direct immune responses [129]. Treg cells widely proliferate in an antigen-specific manner and can respond to both self and foreign peptides. Cytokines released by Treg cells are very important to limit uncontrolled immune responses at environmentally exposed surfaces such as gut. These special T cells play a key role in the maintenance of self-tolerance, therefore preventing autoimmune and inflammatory diseases [129]. Treg cell deficiency results in an effector T-cell response and IBD. This response is driven by reactivity against microbial antigens. Treg cells suppress inflammation through diverse mechanisms including release of inhibitory cytokines such as IL-10, transforming growth factor β (TGF-β), and IL-35 [130]. IL-10 deficient mice maintained in conventional conditions, develop enterocolitis through IL-23 and the Th17 pathway [131, 132].

Much of the current literature on animal models of IBDs pays particular attention to possible links between gut microbiota and mucosal immune responses. Such studies have shown that germ-free mice have an attenuated mucus layer indicating that intestinal microbiota are important in the intensity and function of the mucosal layer [133]. Previous studies have shown that both humans and mice normally tolerate autologous microbiota and the breakdown of this tolerance is associated with chronic intestinal inflammation [134]. It is likely that potential pathological responses to the component of intestinal microbiome, which are restrained by immunoregulatory controls, do occur and when this immune constraint is breached, it modulates the inflammatory response [135]. Also, it is possible that alteration in the composition of gut microbiome toward mainly microbial imbalance and maladaptation of gut microbiota (dysbiosis) disturbs the interaction between the immune system and microbiome and ultimately leads to overemphatic immune responses. These may motivate inflammatory disorders both in and beyond the intestine [136]. Additional observations that suggest possible links between intestinal microbiome and immune response modulation in IBD patients are based on clinical evidence in management of IBD such as patient responses to antibiotic therapy [137–139] and faecal diversion [140–142]. Other investigations identified that IBD patients have higher titres of antibodies against commensal bacteria compared to healthy individuals [143]. Clinically and endoscopically, the distribution of the inflammatory lesions of IBD is more pronounced in the areas of the gut with higher concentrations of bacteria.

### Intestinal Microbiome

#### Introduction

Immediately after birth, environmentally exposed surfaces such as skin, respiratory tract, mouth, vagina and gut are introduced to and colonised by foreign microorganisms [144]. A large and dynamic community of different bacteria is considered a natural inhabitant of the human gut with well-documented effects on human physiology and pathology arising from the interaction between resident bacteria and the mucosal immune system, though the nature of this symbiosis is not very well understood. It has been suggested that the products of gut microbiome play a big role in intestinal health and homeostasis [145]. Impaired gut microbiota function or composition (dysbiosis) can compromise intestinal function and contribute to intestinal pathology. Gut microbiome is a complex and rich ecosystem that supplies multiple levels of intercellular signaling among host and diverse consortia of bacteria, archaea, fungi, protozoa and viruses. It has been estimated that the human gut microbial cells collectively make up to 10 fold of human cells (almost 10^14^ bacteria/g feces or 100 trillion microbial cells in the human gut) [146–148] and across more than 1,200 distinct microorganisms with more than 7,000 strains [147, 149] (mostly in the large intestine). This community of microbes encoded 100-fold more genes than human genome [144, 150, 151]. The bacterial load is the lowest in upper GI tracts (stomach 0–10^2^, duodenum 10^2^, proximal ileum 10^3^) and gradually increases by 10^7^–10^8^ in distal lumen (terminal ileum) [152–154]. This microbial portfolio can be affected by factors such as genetics, birth route, diet, hygiene, psychological distress, environment and lifestyle, infection and medications especially antibiotics. There are several factors that control the prevalence of bacteria in different parts of the GI tract, such as pH, peristalsis, redox potential, bacterial adhesion, bacterial cooperation, mucin secretion, nutrient availability, diet, and bacterial antagonism [155]. It appears that the cross talk between gut microbiota (composition and their products) and cells of the innate immune system in the gut has led to beneficial cooperation, which has established tolerance toward the commensal microbial community in the gut and is thought to out-compete pathogens. The gene products and metabolic output of the gut microbiota play a big role in dynamics and favourable relationship and beneficial interaction within gut microorganisms themselves and with the host. This relationship is important in sustaining the survival of gut microorganisms in their competitive battle with each other which may have adverse effects on human health [156].

Important roles of intestinal microorganisms in the colonic physiology include their influence on epithelial cell growth and differentiation, absorption (mainly calcium, magnesium and iron) [157] and their metabolites which can be used by the host. Gut-bacteria metabolism accounts for the conversion of many substances into metabolites that can be absorbed and used by the host, including energy and vitamin synthesis [133]. One critical class of metabolites are the short-chain fatty acids (SCFA): propionate, butyrate, and acetates. SCFA are produced through fermentation of undigested carbohydrates by commensal bacteria under the anaerobic condition in the large intestine and through binding to G-protein coupled receptor 43 (GPR43) can be used in selected organs including intestine, adipose tissue and immune cells [158]. SCFA are negatively charged radicals (anions) in the colon and are important as energy sources for proliferation, growth and differentiation of colonocytes as well as crypt cell differentiation into three lineage (enterocytes, enteroendocrine cells and goblet cells) that migrate to adjoining villi [133, 159, 160]. Butyrate is mainly consumed by colonic epithelial cells and acetate presented systemically [159, 161]. The role of these SCFAs, their signal to gut receptors (Free fatty acid receptor 2–FFAR2, and receptor 3 expressed by enterocytes, enteroendocrine cells and mast cells) [158, 162] and their influence on appetite control and food intake [163] as well as their anti-cancer (especially for butyrate)/anti-inflammatory properties [164] have been rapidly growing areas of research. The increasing promotion of probiotics and prebiotics to assist human health shows recognition of a potential role for beneficial and harmful microbial populations in the aetiology, prevention and control of disease processes [165–168], although data supporting their role in health remain scant.

Most of the initial studies on human gut microbiota were based on standard culture techniques [169] and molecular analysis [170, 171], but more than 90% of the environmental microbes cannot be easily cultured [172], so microbial ecologists developed other culture independent approaches to study microorganisms including using the 16S ribosomal RNA gene (16SrRNA) as a phylogenetic and taxonomic indicator for members of microbial communities [173–175], with further development of novel gene sequencing technologies and availability of powerful bioinformatics analysis. 16SrRNA was the first revolution in culture independent approach in the late 1970s and as a phylogenetic marker to explain microbial structure and diversity [176]. Later metagenomic investigations revealed that the variability of abundance of microbial species in individuals can greatly affect identification of the possible common microbial signature such as prominent clusters comprise some of the most abundant gut species. Such gut species include members of the Firmicutes (∼64%) the Bacteroidetes (∼23%), the Proteobaceria and the Actinobaceria [149, 154, 177, 178].

The composition of the gut microbiota is shaped by the influence of individual genetics [179], as do other intrinsic and extrinsic host factors, such as diet, drugs, xenobiotics and diseases [180]. The interactions between gut microbiome and host are mutual among individuals but surely not identical. This discrete microbial-host interaction resulted to distinct microbial profile that differentiates one individual from all others [181]. Therefore, in spite of the phylogenetic diversity only a subset of identifiable microbial niches with overlapping functional properties and products but not microbial species, were shared between all individuals which refutes the existence of a “core” microbiome [154, 165, 182, 183].

Although there is evidence showing extensive α and β diversities of human associated microbes [184] and substantial diversity within healthy individuals (mainly among infants) [144, 182, 185, 186], sequence based methods (including 16SrRNA) have confirmed that at phyla level, human gut microbiome is less diverse comparing to other ecosystems (such as soil, ocean water); only limited numbers of bacterial domains are detected in human stool or gut mucosal samples dominated by two bacterial phyla: “Firmicutes” and “Bacteroidetes” (more than 90%) [187, 188]. Such analysis also highlighted other bacterial divisions identified in distal gut as Proteobacteria, Actinobacteria, Fusobacteria and Verrucomicroba [187]. Nonetheless in the lower taxonomic levels (species) there are considerable variations in human gut microbial composition [187, 188].

#### Gut Microbiome and IBDs

It has long been proposed that gut bacteria play an important role in the pathogenesis of IBD through their direct interaction with the intestinal mucosa. As already mentioned, IBDs are characterised by inappropriate immune upregulation in genetically susceptible patients and it seems that gut microbiota are the target of this inapt immune response either due to loss of tolerance toward commensal bacteria or secondary to an altered microbial diversity and/or function [189]. Animal studies have emerged as powerful platforms for studying the role of gut microbiota in the development of the immune system and immune response. Mombaerts and colleagues showed that transgenic mice expressing aberrant T cell receptors spontaneously develop colitis in response to normal intestinal microbiota [190]. Many subsequent studies present convincing evidence confirming the involvement of the enteric bacteria in pathogenesis of IBD. A range of bacteria are stated to have aggressive or protective functions in intestinal inflammatory disorders such as Crohn’s disease; for example phlogistic effects of adherent-invasive *Escherichia coli* [191] and protective effects of Faecalibacterium prausnitzii [192]. In both clinical and experimental cases of IBDs, the abundance of Enterobacteriaceae [193], and Fusobacteria [194] are increased. Other studies have convincingly shown that diversion of the fecal stream induces clinical remission and can prevent recurrence of CD, while infusion of intestinal contents to excluded ileal segments reactivates mucosal lesions [195, 196]. Organ culture of inflamed mucosal samples from patients with active IBD (CD and UC), have confirmed that co-culture with non-pathogenic *Escherichia coli* strains strongly stimulates release of proinflammatory cytokines (IL-6, IL-23p19, IL-12p35, IL-17F, IFN-γ, TNF-α), proinflammatory chemokines (IL-8, CXCL-1 and CXCL-2), which activates the inflammatory cascade, whereas the level of TNF-α and IL-6 was significantly lower in healthy controls or when non-pathogenic *E coli* strains were co-cultured with *Lactobacillus* casei [197, 198]. Studies on mice, rats and guinea pigs have shown increasing evidence that antigens derived from communal bacteria, regulate the immune response as the absence of the healthy community of bacteria in these studies is correlated with non-appearance of intestinal inflammation [199]. To examine the integrated impact of gut microbiota in the pathogenesis of IBDs, it is important to incorporate microbiome data with other data related to immune modulation, genetics, psychological and physiological risk factors.

### The Autonomic Nervous System–Connecting the Brain and Body

#### Introduction

The autonomic nervous system (ANS) is a complex regulatory and signaling system connecting the brain and body. Its function is to maintain homeostasis and facilitate self-regulation and adaptation [200]. The ANS consists of a parasympathetic and a sympathetic division. The parasympathetic nerves subserve vegetative and restorative functions and rest-and-digest activities. The sympathetic system enables energy consuming processes during activity or stress known as the “fight or flight” response to increase endurance and ensure survival. There is a growing body of literature that recognises the role of ANS in chronic disorders as disparate as chronic fatigue syndrome, post-infective fatigue syndrome [201, 202], ischaemic heart disease [203], and chronic depression [204]. In the study of IBDs, a much debated question is whether any disturbance in autonomic function and in bidirectional communications between CNS and gut could establish vulnerability toward persistent functional syndromes and/or chronic gut inflammation [205, 206].

#### ANS, the Key Body-Brain Connection

The human brain constantly receives signals from the outside environment through different sensory mechanisms [205, 207]. In addition, internal sensory systems collect vital information about the physiological state of multiple physiological microenvironments including any disturbances in gut homeostasis, inflammation, and tissue damage [208–210]. This information is relayed by several afferent neural and endocrine pathways to both autonomic/homeostatic brain centres and to higher limbic and cortical regions. This dynamic processing of information and consecutive cortical integration provides the brain with “conscious awareness” of the internal physiological state-a process known as interoception [208]. The primary interoceptive representation is located in the right anterior insula [211]. The brain’s reaction to such information is to adjust descending autonomic regulation, and to alter emotions and motivated behaviour to ensure adaptive survival [208]. The development of human autonomic response pathways throughout evolution has favored sympathetic dominance to increase the chance of survival and adaptive response [212, 213]. Active inhibition executed by prefrontal cortical circuits restrains sympathetic signaling during rest and recovery [214], and through this top-down modulation, it facilitates parasympathetic (vagal) activity. Stressful conditions such as perceived and prolonged psychosocial challenges and adversities, or physiological stressors, such as sickness or inflammation, reduce the prefrontal inhibitory control over sympatho-excitatory circuits, leading to sympathetic dominance and loss of vagal tone [215]. The aim behind this autonomic shift is to enable endurance under challenging conditions. However, prolonged sympthetic hyperactivity engenders a defensive physiological state that lacks dynamic flexibility and this has been associated with poor sleep, depressed mood, anxiety and inflexible cognitive functioning; as well as a wide variety of medical and psychiatric conditions [213, 215–219].

#### ANS Control in Gut Inflammation–Relationships Between Central and Peripheral Nervous Systems in IBDs

The ANS maintains constant communication between the brain and the gut [220]. Evidence from animal and human studies has revealed a set of physiological, behavioral, motivational, and cognitive changes accompanying activation of the immune system, such as malaise, fever, hyperalgesia, increased need to sleep, disturbed mood and impaired concentration and learning. Together these are known as the acute sickness response-a highly organised and evolved disease fighting strategy to increase survival [221–223]. Study of the sickness response is an important key to understanding the role of the brain in sickness and recovery, including intestinal inflammation. Further investigations into the mechanisms of the sickness response at the molecular level revealed that sickness-related changes are due to the effects of proinflammatory cytokines (IL-1; including IL-1α and IL-1β, IL-6 and TNF-α) on the brain [224, 225]. The gut environment fosters a broad network of enteric neurones, innervating the gut in close proximity to gut homing immune cells. This complex innervating network includes myenteric plexus that innervates longitudinal and circular smooth muscles in muscularis externae which is also the home for immune effector cells [226]. Submucosal plexus secretomotor neurons innervate goblet cells and enterocytes [226]. The afferent vasodilator neurones and sympathetic vasoconstrictive nerves, coat submucosal vasculature and regulate the function of these tissues in response to mechanical (stretch of the wall, mucosal layer damage) or chemical (bacterial products, toxins), stimuli [227, 228].

Inflammatory bowel diseases are associated with upregulation of the intestinal immune response with increased proinflammatory cytokines and chemokines during the active phase of the disease [3]. Clinical evidence has indicated a possible relationship between ANS function and inflammatory activities in IBDs. The ANS, together with the enteric nervous system (ENS) have important roles in gastrointestinal motility, secretion and mucosal immune response [229]. Furthermore, research in animal models of intestinal inflammation has addressed the role of ANS as an important modulator of inflammatory activities [205]. Such studies highlighted the position of the sympathetic and parasympathetic nervous system in intestinal inflammation, including altered ANS activity in UC patients [230, 231] and disparity in vagal tone in CD and IBS patients [232]. In the presence of gut inflammation or injury, peripherally released cytokines can influence the brain via hard-wired, fast-tracked pathways (from afferent nerves innervating the inflamed site) and via slower endocrine pathway (e.g. cytokines originating in the choroid plexus and circumventricular organs, and their volume transmission into the brain through blood brain barriers) [224, 233]. These two pathways act independently of each other [234]. Endocrine afferent transmission of inflammatory signaling to the brain is through a slower trail and by the production of molecular intermediates including prostaglandins in response to an increase in inflammatory cytokine levels. This pathway also activates the hypothalamus-pituitary-adrenal (HPA) axis, which in return releases anti-inflammatory and immunosuppressive proteins–the glucocorticoids. Neuroanatomical studies of gastrointestinal afferent neurons show that there are three neural pathways transmitting signals from the gut to the CNS including vagal afferent signals (vagal afferent fibers are densely concentrated in upper GI tract), pelvic afferent signals (mainly from the colorectal regions) and splanchnic afferents [235]. One fast tract afferent neural pathway is through sensory neurons of the vagus nerve innervating organs of the abdominal cavity that express receptors for proinflammatory cytokines. The vagus nerve (the body’s longest nerve with 20% efferent and 80% afferent fibres) is a component of the neuro-immune axis through its efferent and afferent fibres. It is the chief constituent of the parasympathetic nervous system (PNS) and connects the CNS to major visceral organs (including lungs, heart, and gut), [220]. During intestinal inflammation and the release of proinflammatory cytokines, visceral information is transferred to the nucleus tractus solitarius (NTS) in the medulla oblongata in CNS either through activated vagal afferent nerves or through splanchnic and pelvic nerve afferents to the thoracolumbar and sacral spinal cord [220, 236, 237]. The brain’s response to intestinal inflammation is translated into action potentials in the efferent vagus nerve which function to inhibit the production of proinflammatory cytokines [238]. This anti-inflammatory property of vagus nerve is through efferent cholinergic anti-inflammatory pathways. This circuit fundamentally includes the interaction between neurotransmitter acetylcholine and its alpha7 nicotinic acetylcholine receptor subunit expressed on monocytes, macrophages and other cytokine producing cells [239], restraining cytokine release, reducing production of TNF-α by macrophages (by blocking α7nAChR) to attenuate inflammation [240–245] and preventing tissue damage in the gut [246–248].

The sympathetic division enables both the stimulation of proinflammatory mediators (in the acute phase of inflammation) and anti-inflammatory properties (in the chronic phase of inflammation) [249, 250]. The sympathetic nervous system (SNS) and its neurotransmitters-the catecholamines, modulate GI movement, reduce intestinal motility, control secretion in GI tract (through its postganglionic sympathetic innervations), induce vasoregulation [250–252] and potentiate inflammatory cascades in the presence of immunogenic stimuli [232, 253–256]. Sympathetic innervating axons are in close proximity to GALT homing T cells, B cells, macrophages and mast cells [257]. Mast cells homing in intestinal mucosa are important mediators in inflammatory processes by releasing a variety of cytokines and chemokines, inducing intestinal hyper-permeability and activating inflammatory cascades [249, 250, 258, 259]. In the presence of an immunogenic stimulus, the sympathetic signaling pathways act to upregulate the host immune response against the stimuli through activating immune and enteroendocrine cells, and this is frequently accompanied by heightening visceral sensation, [230, 260–262]. Some clinical studies have suggested that the presence of such systemic inflammatory disorder leads to the development of autonomic dysfunction in CD and UC patients [263–267]. In addition, it is possible that consistent sympathetic hyperactivity in the presence of chronic inflammatory disorders such IBDs initiate autonomic dysfunction as seen in chronic depression or chronic heart diseases [250, 268]. Such findings broadly support the interplay between the intestinal immune system and inflammatory cell activity with the autonomic and enteric nervous system and the CNS [269]. Despite mixed results from several clinical studies in regard to a possible causal role for autonomic dysfunction in the disease aetiology [230, 267], all have shown evidence of autonomic impairment in chronic inflammatory disorders [205].

#### Measures of Autonomic Reactivity

Experimental studies enhanced our understanding on how physiological stimuli, affect autonomic regulation and dynamically regulate heart rate through vagal and sympathetic efferent nerves [270, 271]. In healthy subjects the electrical activity of the sinoatrial node located at the posterior wall of the right atrium initiates each beat of the heart. Due to the unstable membrane potential of the myocytes located in this region, action potentials are generated periodically at a fairly constant frequency. This relatively constant frequency-generated by the autorhythmicity of the sinoatrial node-is modulated by many factors that add variability to the heart rate signal at different frequencies. These frequencies are affected by temperature regulations, endocrine systems, cardiac sympathetic and parasympathetic nerve activity, and respiratory rhythm (Task force of European Society of Cardiology and The North American Society of Pacing and Electrophysiology, 1999). Physical activity and/or experiencing stressful events involve a shift toward sympathetic dominance and a concomitant increase in the heart rate. A number of cardiac autonomic measures such as the time-to-recovery of resting heart rate after stress and certain parameters of beat-to-beat heart rate variability (HRV) are considered valid scientific tools to quantify autonomic balance and by inference the inhibitory influence of prefrontal cortical circuits, through parasympathetic (vagal) pathways, on sympathetic nerve activity [272]. This is supported by evidence from clinical studies which has consistently identified low HRV as an indicator of diminished parasympathetic control and a marker for increased risk of morbidity and mortality [273, 274]. Such evidence implicates autonomic dysfunction as specified by decreased HRV together with increased levels of systemic inflammatory markers (CRP and IL-6) [275] in heightened risk of cardiovascular morbidity [276]. Additionally low HRV has been linked to psychosocial variables and perceived stress [277] as well as clinical depression [278] and chronic fatigue syndrome [201]. In patients with IBDs, assessments of beat-to-beat HRV during autonomic arousal in response to stressors has similarly suggested impaired autonomic functioning [229, 279]. Evidence from examining HRV and autonomic reactivity in IBD patients in remission has suggested autonomic recovery indicated by a swing toward stronger parasympathetic responses, lower systemic inflammatory activity (CRP) and sympathetic withdrawal [229, 280].

### Psychosocial Factors

#### Introduction

An organism’s response to environmental stressors–which may constitute real or perceived threats to body integrity and homeostasis–consists of activating a complex range of pathways, involving the endocrine, nervous, and immune systems [281]. The short-term fight-or-flight stress response enables survival by shifting to sympathetic dominance, which stimulates the neuro-endocrine and cardiovascular systems to favor endurance and increase survival [282]. This response includes physiological and behavioural changes such as increased awareness, heightened arousal, increased cardiovascular tone, increased respiratory rate, improved analgesia [283], immune activation and inhibition of vegetative tasks (such as feeding, digestion, reproduction) [63]. Inappropriate activation of the stress response, which is common to many psychological disorders such as anxiety, has been linked to a wide array of pathological conditions including autoimmune disease, hypertension and affective disorders, [63]. A body of literature has been published on possible associations between psychological state and disease activities in chronic inflammatory diseases such as IBD; so far, however, there has been little consensus on whether psychological distress should be considered a cause or effect in IBDs or both.

#### Inflammatory Bowel Diseases are Associated with Clinically Significant Psychological Manifestations

There have been many studies in IBD documenting an association between the onset of IBD with recent stressful life events and suggested any perceived psychological distress even not related to disease activity can also influence the course of the disease [65, 284, 285]. Such studies also examined the effects by the disease on patients’ psychological state. IBDs are chronic debilitating disorders which may affect many aspects of the sufferer’s life and can add to the psychological burden including high levels of perceived stress [286], negative mood and depression [287] and anxiety compared to the healthy population [288]. The prevalence estimate of both depression and anxiety were higher in IBD patients -even among patients in remission-than what was expected in general population [289–291]. Studies have shown associations between symptoms of depression and anxiety with more severe IBD symptoms, more episodes of relapse in IBD patients [292], and higher rates of hospitalisation [293]. On the other hand, observed co-morbidities of high anxiety and depression irrespective to the disease itself in IBD patients have raised the idea of a causal relationship between these two conditions and IBD symptoms [294]. Two national representative population-based Canadian surveys [295] reported that IBD patients have 3 times higher depression rates than the healthy population. The same results were identified in the study by Mikocka-Walus et al, especially in patients during active phases of the disease [296]. A systematic review on primary studies examining depression and anxiety in IBD patients between years 1967–2014 [73] suggested that nearly one-fifth of IBD patients have shown depressive symptoms and approximately one-third were experienced anxiety symptoms. Such systematic reviews similarly suggested that patients with active disease had significantly higher prevalence of depression (40.7%) than those in remission (16.5%) [290, 291] and anxiety symptoms were more prevalent in active disease than those who were in remission. Furthermore, anxiety and depression were more common in CD patients compared to those with UC [291].

However, there were mixed and conflicting results to determine whether depression and anxiety developed before or after onset of IBD [297, 298]. Such mixed results might have been simply driven from the methological differences in study design with limitted controlling for confounder or biases including recall biase (retrospective Vs prospective study design, short term assesment Vs longitudinal assessment, one off data collection Vs time series examination), models for data collection (qualitataive Vs quantitative) and/or study population/cohort under observation (which may not have been completely representative) i.e. some studies examined both CD and UC patients in one IBD cohort and some examined the psychological states in IBD patients irrespective of the disease activity (active Vs remission) or disaese phenotype/behavior. Also, such variations might have been caused by differences in healthcare system, community awareness of the disease, differences in lifestyle, environmental factors, access to medication in the countries studied.

Many IBD symptoms may be due to stress-induced changes in gastrointestinal function, immune regulation [297] and by affecting the composition of gut microbiota as a result of reducing the microbial diversity and increasing the ratio of inflammation-promoting bacteria [253]. Evidence from studies of experimental stress in animal models of colitis supports the association between psychological distress and IBD disease activity; showing a model of psychoneuroimmunology in interaction between immune function, nervous system and gut microbiota composition and function [253]. Studies also show that psychological distress and IBD disease activity consistently predict lower quality of life measures [296, 299]. IBD patients who show symptoms of anxiety and depression have reported lower quality of life [300, 301] and the disease course is more sever in those IBD patients with depression [64]. It is important to clinically identify psychological distress to ensure that patients are receiving IBD-specific psychological support as part of a patient focused model of care.

#### Neuropsychological State and IBDs-a Rapid Response

The difficulty in examining the relationship between psychological states and the course of IBDs is mainly related to whether the psychological distress is the product of the IBDs as a chronic inflammatory and debilitating disorder or a predisposing and contributing risk factor for the onset and/or maintenance of the diseases. Both the autonomic nervous system and HPA axis are actively involved in the inflammatory responses in human [302]. Moreover, both can be stimulated by psychological and physical stimuli, which in turn suggests that neuropsychological states can affect the progress and intensity of chronic inflammatory disorders such as IBD. This also indicates that the up- or down-regulation of the HPA axis and ANS response during chronic inflammation may both affect the physiological response to stress.

As both psychological factors and autonomic nervous system are integrated and can influence patients’ effective adjustment to the course of the disease, both need to be considered in IBDs [303]. ANS regulates bidirectional neural cross talk including via autonomic afferents to the CNS, which in turn adjusts efferent signaling of the ANS [304, 305]. Neural circuits in higher brain regions including the prefrontal cortex, the hippocampus, and the amygdala can also control these efferent regions [289, 306] and modulate gut function, in addition to influencing the emotions, cognition and behaviour [307, 308]. Although the literature suggests the presence of an autonomic dysfunction in IBD patients, the extent of such autonomic dysfunction depends on patients’ coping strategies and emotional states. The mechanism of this dysfunction is not very well understood [303].

#### Hormonal Response and Psychological Stress

Psychological distress can threaten homeostasis therefore homeostasis is re-established by various physiological and behavioral adaptive responses. Neuroendocrine hormones have major roles in the regulation of both basal homeostasis and responses to threats and are involved in the pathogenesis of diseases. The stress response is mediated centrally, as well as in peripheral tissue through peripheral mediating systems. While rapidly responding ANS mediators of the stress response have been outlined above, the principal sites that modulate hormonal stress responses are the hypothalamus (the paraventricular nucleus-PVN), the anterior lobe of pituitary gland, and the adrenal gland (the hypothalamic-pituitary-adrenal axis), as well as sympathetic adreno-medullary circuits, causing the release of adrenaline [309, 310]. The principal regulator of the HPA axis is corticotropin releasing factor (CRF), secreted by the hypothalamus in response to environmental stimuli and stress. The hypophysial portal vessels transfer CRF to the anterior pituitary gland and induce the release of adrenocorticotropic hormone (ACTH) after binding to CRF receptors [311]. ACTH reaches the adrenal cortex by the systemic circulation and stimulates the synthesis and release of glucocorticoids. Glucocorticoids are a major subclass of steroid hormones and downstream effectors for HPA axis that modulate the body’s physiological changes [300]. Glucocorticoids regulate metabolic, cardiovascular, immune, and behavioural processes [283]. These processes are accountable for adaptive changes, but depend on the type, period, and intensity of the stimuli as well as extent of the activation of HPA axis, with excessive or inadequate activation possibly engendering a pathologic impact on the body and lead to a host of behavioral and somatic pathological conditions [312]. Psychological and emotional stress can affect the sensory and secretory function of digestive system [313] and may alter the cholinergic nervous system, function of intestinal mucosal mast cells and subsequently increases in intestinal permeability [314], which is commonly observed in the active phase of IBD.

### Conclusion

In the last few decades, there has been a surge of interest in studying the factors affecting the onset and severity of IBDs. Although the etiology of the IBDs is not very well understood, it is increasingly clear that multiple factors including genetic, environmental, psychological, autonomic, immunological, and gut microbiota interact and contribute to the disease manifestations and their persistence.

Genetic studies of IBD have highlighted the role of genetic susceptibility and its likely co-dependence with other mediators such as environmental, immune, and microbial factors. Recently, a considerable body of literature has accumulated relating to the effect of psychological state and IBDs, indicating higher level of depression, stress and anxiety experienced by sufferers especially in the active phase of the disease. Psychological distress is an inseparable element of chronic disorders; hence there is still a need of comprehensive qualitative and quantitative research to investigate the causality of psychological factors and IBD activity. IBDs are characterised by prolonged immune activation in the gut in association with a breakdown in regulatory constraint, and dysbiosis. It is not clear though, if immune activation is a product of dysbiosis or the loss of tolerance in innate and adaptive immunity in the inflamed gut environment. Although a shift in the microbial diversity and IBD phenotype has been addressed in many clinical and experimental studies, thus far no evidence exists to confirm causation.

The significant association between a maladaptive autonomic reactivity with the onset and manifestations of some chronic disorders has suggested possible links between the ANS and IBDs disease activity. It is important that research examining a role for vulnerabilities and individual differences of IBD patients incorporates longitudinal assessments of biological and psychological factors and their temporal trajectory (including in remission and relapse) with the aim of characterising the risk factors and interdependence of these major factors in the course of the disease. Previous published studies were limited in the number of risk factors examined, or they lacked longitudinal analysis of such factors contributing to IBD disease activity [61, 291, 315, 316].

## Glossary

ACTH: adrenocorticotropic hormone
ADHD: attention deficit hyperactivity disorder
ATH: adrenocorticotropic hormone
ANS: autonomic nervous System
CD: crohn’s disease
CRF: corticotropin releasing factor
CRP: C-reactive protein
DASS: depression, anxiety, stress scale
DMI: depression in medically ill
DS-NA: type D personality scale of negative affectivity
DS-SI: type D personality scale of social inhibition
ENS: enteric nervous system
F-calpro: faecal calprotectin
GI: gastrointestinal
HF: high frequency
HRR: heart rate reactivity
HRQOL: health related quality of life
HRV: heart rate variability
IBD: inflammatory bowel disease
IL: interleukin
IFN- Ɣ: interferon gamma
LF: low frequency
LIMBO Study: longitudinal course of inflammatory bowel disease study
MDP: muramyl dipeptide
NOD2: nucleotide-binding oligomerization domain-like receptors 2
PAMPS: pathogen associated-molecular patterns
PNS: parasympathetic nervous system
PRR: pattern recognition receptors
PSQ: perceived stress questionnaire
PSQI: pittsburgh sleep quality index
PVN: para ventricular nucleus
QOL: quality of life
RMSSD: root mean square of the successive differences between N-N intervals
ROS: reactive oxygen species
SDNN: standard deviation of all normal R-R (N-N) intervals
SNS: sympathetic nervous system
TGF-β: transforming growth factor β
TLR: toll like receptors
TNF-α: tumour necrosis factor α
UC: ulcerative colitis
